# Performance of the STS Risk Score in Predicting Outcomes in Impella-Supported High-Risk Percutaneous Coronary Intervention: Insights From the PROTECT III Study

**DOI:** 10.1016/j.jscai.2026.105448

**Published:** 2026-06-11

**Authors:** Batla Falah, Julia Thompson, Yousuf Shah, Tayyab Shah, David J. Cohen, Guido Ascione, Pedro Melo, Björn Redfors, Mir Babar Basir, Wayne B. Batchelor, Alejandro Lemor, Alexander G. Truesdell, Cindy L. Grines, William W. O’Neill

**Affiliations:** a Cardiovascular Research Foundation, New York, New York; b Department of Internal Medicine, Strong Memorial Hospital, Rochester, New York; c Division of Cardiovascular Medicine, Hospital of the University of Pennsylvania, Philadelphia, Pennsylvania; d St. Francis Hospital, Roslyn, New York; e Department of Population Health Sciences, Weill Cornell Medicine, New York, New York; f Department of Molecular and Clinical Medicine, Gothenburg University, Gothenburg, Sweden; g Department of Cardiology, Sahlgrenska University Hospital, Gothenburg, Sweden; h Division of Cardiology, Henry Ford Hospital, Detroit, Michigan; i Inova Medicine Service Line and Schar Heart and Vascular, Inova Health System, Falls Church, Virginia; j Department of Cardiology, University of Mississippi Medical Center, Jackson, Mississippi; k Virginia Heart/Inova Schar Heart and Vascular, Falls Church, Virginia; l Department of Cardiology, Northside Hospital Cardiovascular Institute, Atlanta, Georgia; m Center for Structural Heart Disease, Division of Cardiology, Henry Ford Health System, Detroit, Michigan

**Keywords:** cVAD registry, high-risk percutaneous coronary intervention, major adverse cardiovascular and cerebrovascular events, PROTECT III, Society of Thoracic Surgeons Risk Score

## Abstract

**Background:**

Patients undergoing high-risk percutaneous coronary intervention (HRPCI) with Impella often present with similar or worse anatomical complexity than surgical revascularization candidates, yet the performance of surgical risk models in this setting is unknown. We evaluated Society of Thoracic Surgeons (STS) score performance in predicting outcomes in the PROTECT III study.

**Methods:**

PROTECT III enrolled 1237 patients undergoing elective or urgent HRPCI with Impella support from March 2017 to March 2020. Patients were stratified into low (STS <4), intermediate (≥4 to ≤8), and high (>8) risk categories. Primary endpoints were 30-day major adverse cardiovascular and cerebrovascular events (MACCE) and mortality. Model discrimination was evaluated with receiver operating characteristic analysis; calibration was assessed with observed-to-expected (O/E) ratios and Hosmer–Lemeshow testing. Secondary endpoints included 90-day MACCE and 1-year mortality.

**Results:**

Of 1237 patients, 728 patients were low-risk, 316 intermediate, and 193 high-risk based on STS score. Higher STS risk was associated with a higher number of comorbidities and more left main and multivessel disease. At 30 days, MACCE rose with risk (low 5.1%, intermediate 10.1%, high 17.6%; *P* < .001), driven by mortality (3.5%, 8.6%, 16.8%; *P* < .001). Patterns persisted at 90 days. The STS score showed good discrimination for 30-day percutaneous coronary intervention mortality (C-index 0.72; 95% CI:, 0.66-0.77) with acceptable global calibration (Hosmer–Lemeshow *P* = .13), comparable to the HRPCI-specific risk score. However, it underestimated mortality at 30 days (O/E 1.23), suggesting that existing scores do not capture the high-risk nature of this population.

**Conclusions:**

The STS score provides reasonable discrimination in Impella-supported HRPCI but poorly predicts event rates, particularly underestimating mortality. These results underscore the need for modification of existing models and validation of HRPCI-specific tools.

## Introduction

The proportion of patients undergoing high-risk percutaneous coronary intervention (HRPCI) is increasing in part due to aging populations, delayed presentation, and a growing burden of comorbidities.^1^, ^2^, ^3^ Nearly half of these procedures involve left main (LM) or multivessel disease.^4^^,^^5^ Many of these patients meet anatomical criteria for coronary artery bypass grafting (CABG) but are deemed ineligible for or decline surgery due to high procedural morbidity and mortality risk or frailty.^6^^,^^7^

In this context, percutaneous left ventricular assist devices (pLVADs), such as the Impella, can enable safe and feasible HRPCI in selected patients by providing hemodynamic support during complex revascularization in high-risk patients.^3^^,^^8^^,^^9^ However, despite the physiologic benefits, Impella use remains associated with potential complications, most notably bleeding, vascular injury, hemolysis, and acute kidney injury.^10^^,^^11^ As these risks decline with improved patient selection and operator and team experience, individualized risk stratification remains a critical component of preprocedural patient counseling and procedural planning.

Current guidelines advocate for the use of validated risk scores to assist in preprocedural decision making for coronary revascularization.^12^^,^^13^ Recently, a dedicated HRPCI risk score was developed to predict in-hospital major adverse events among patients undergoing Impella-supported PCI.^14^ This score demonstrated good discriminative performance but has limited external validation and lacks comparison to broader, widely adopted risk models.

The Society of Thoracic Surgeons (STS) risk score is a well-established and extensively validated tool for estimating 30-day mortality and morbidity after CABG.^15^^,^^16^ It incorporates comorbidities, clinical status, and procedural variables to objectively quantify surgical risk and has been routinely used in surgical evaluations since 2003. Although the STS score was not developed for PCI, it is often calculated in patients with complex coronary disease who are referred for surgical consultation even when PCI is ultimately pursued. This is particularly relevant in patients undergoing HRPCI with pLVAD support, as they frequently present with multivessel disease or LM involvement, conditions historically considered for surgery.

Despite its widespread use in guiding CABG decisions, STS risk score performance in the HRPCI population has not been systematically evaluated. Given the overlap in clinical and anatomical complexity between surgical and HRPCI candidates, we sought to assess the utility of the STS score in predicting outcomes in patients undergoing Impella-supported HRPCI and to compare its performance with the recently developed HRPCI risk model.

## Materials and methods

### Study design, population, and oversight

The design of the PROTECT III study has been previously described.^17^ Briefly, PROTECT III is an US Food and Drug Administration-audited, single-arm, observational study that prospectively enrolled 1237 patients across 46 centers in North America between March 2017 and March 2020. Enrolled patients underwent elective or urgent HRPCI procedures (ie, without cardiogenic shock) with an Impella 2.5 or Impella CP pLVAD implanted for procedural hemodynamic support. This study is part of the global cVAD registry (NCT04136392), including several separate post-approval studies intended to evaluate the safety and efficacy of Impella mechanical circulatory support use for different cardiovascular conditions. The decision to perform HRPCI, use an Impella device, and the determination of indications for its use were at the discretion of the treating physician and aligned with local standard practices. Patients were enrolled once the decision to use Impella was made prior to or during the index PCI. Cases in which the device was implanted as a bailout after PCI were excluded.

Baseline demographics, echocardiographic parameters, and procedural details were collected before the index procedure. Angiographic data were analyzed by an independent core laboratory (Beth Israel Deaconess Medical Center Angiographic Core Laboratory). All clinical events were adjudicated by an independent clinical events committee through 90 days, and patients were followed to 1 year for vital status assessment.

The study was conducted in accordance with the principles outlined in the Declaration of Helsinki and was approved by the institutional review board or independent ethics committee at each participating site prior to patient enrollment. The sponsor (Abiomed Inc) oversaw data management and source document verification and provided funding to the Cardiovascular Research Foundation and Meducator for independent statistical analyses. The authors had full, unrestricted access to the study data and take full responsibility for the accuracy and integrity of this report.

### Study population and STS risk calculation

This analysis included all patients from the PROTECT III study. The STS predicted risk of 30-day mortality was calculated using the online STS Operative Risk Calculator (version 2.0.5). A Python script (version 3.9) was created to automate the input of each subject’s data into the online risk calculator. The script operated by mapping the assigned variables to the correct location in the calculator for each subject. If information regarding a particular covariate was not available for a specific patient, it was left blank. Data from 50 patients were confirmed manually to validate the accuracy of the methodology used by the Python script.

### STS group definition

After calculation of the STS score for each patient, individuals were stratified into 3 risk categories: low risk (STS <4), intermediate risk (STS ≥4 to ≤8), and high risk (STS >8).

### Study endpoints

The primary endpoints were: (1) 30-day major adverse cardiovascular and cerebrovascular events (MACCE), defined as the composite of all-cause mortality, myocardial infarction, stroke/transient ischemic attack, and repeat revascularization, stratified by STS risk categories (low, intermediate, and high); and (2) the performance of the STS risk score in predicting 30-day mortality, assessed using receiver operating characteristic curve analysis and the observed-to-expected (O/E) event ratio. Secondary endpoints included 1-year all-cause mortality and 90-day MACCE, also compared across the 3 STS risk groups. Detailed definitions of adverse events in the PROTECT III study have been published previously.^17^

### Statistical analyses

Baseline characteristics were summarized with mean ± standard deviations or medians and interquartile ranges for continuous measures and proportions for categorical variables. For comparisons between the study groups, categorical variables were compared using the χ^2^ or Fisher exact test, while continuous variables were compared using analysis of variance or the Wilcoxon rank-sum test. For time-to-first event analyses, event rates were estimated by the Kaplan–Meier method and compared with the log-rank test.

The observed 30-day adverse events (stroke, reoperation, renal failure, and mortality) in the PROTECT III patient cohort were compared with the corresponding event rate predicted by the STS risk score (expected) in 2 ways. First, the O/E event rate ratio was calculated for each outcome along with its 95% CI. Second, we examined the relationship between the observed and expected mortality by dividing patients into deciles of STS predicted mortality and plotting observed versus predicted mortality rates for each decile. Goodness of fit was tested by using *R*^2^ values, and the line of best fit was compared to the line of identity using an F-test for the null hypothesis that the slope is 1, and a *t* test for the null hypothesis that the intercept is 0.

All *P* values are 2-tailed, and *P* < .05 was considered significant for all analyses. Statistical analyses were performed using SAS version 9.4 (SAS Institute Inc).

## Results

### Baseline characteristics

**Figure 1:**
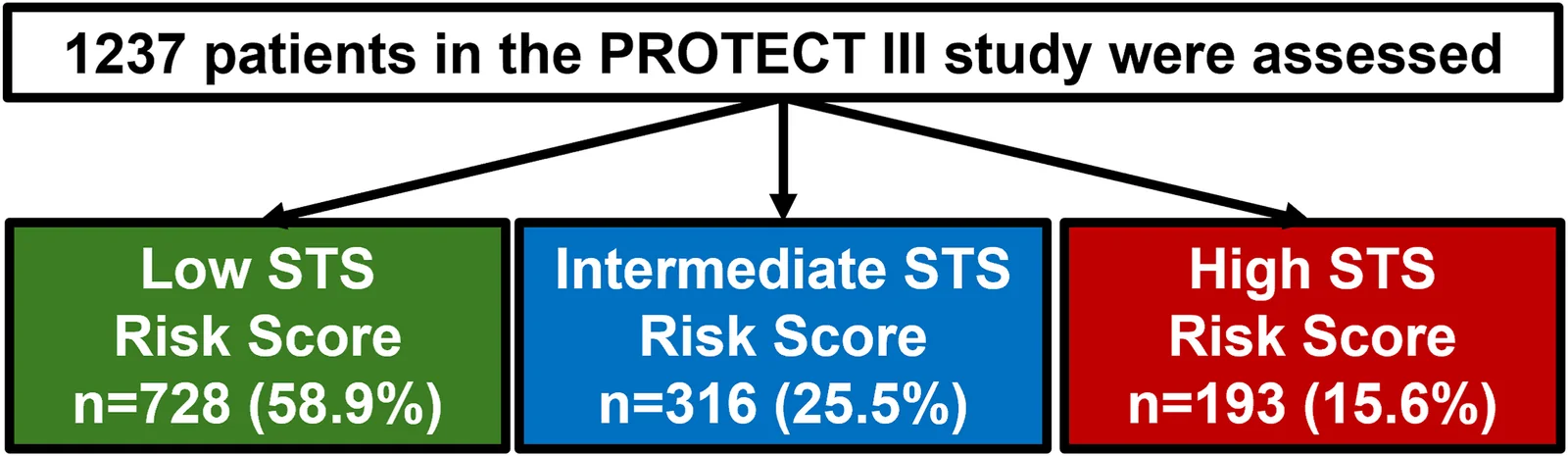
**Study flow chart.** STS, Society of Thoracic Surgeons.

Between March 2017 and March 2020, PROTECT III enrolled 1237 consecutive patients across 46 North American sites, stratified by STS risk as low (<4; n = 728, 58.9%), intermediate (≥4 to ≤8; n = 316, 25.5%), or high (>8; n = 193, 15.6%) (Figure 1). Higher STS category patients were characterized by older age, a lower proportion of men, and a greater comorbidity burden, including more established coronary disease and prior revascularization, peripheral arterial disease, prior myocardial infarction, heart failure with lower left ventricular ejection fraction, and more advanced renal dysfunction with greater dialysis use. In contrast, the prevalence of diabetes, hyperlipidemia, and chronic obstructive pulmonary disease did not differ significantly among groups (Table 1).

**Table 1:** Baseline demographics and procedural characteristics according to STS risk score

Characteristics	STS risk score			Overall *P* value
	Low (n = 728)	Intermediate (n = 316)	High (n = 193)	
Demographics				
Age, y	67.2 ​± 10.8	75.3 ​± 9.3	78.4 ± ​8.5	<.001
Male sex	78.6 ​(572/728)	68.4 ​(216/316)	60.6 (117/193)	<.001
Race				
White or Caucasian	68.8 ​(501/728)	67.4 ​(213/316)	60.6 ​(117/193)	.10
Black or African American	9.6 (70/728)	17.7 (56/316)	14.0 (27/193)	.0010
Asian	2.9 (21/728)	1.9 (6/316)	5.7 (11/193)	.05
American Indian or Alaska native	0.5 (4/728)	0.3 (1/316)	0.5 (1/193)	.88
Native Hawaiian/other Pacific Islander	0 (0/728)	0 (0/316)	0.5 (1/193)	.07
Other race	2.5 (18/728)	4.4 (14/316)	5.2 (10/193)	.09
Body mass index, kg/m^2^	29.1 ​± 6.2 (725)	28.0 ± ​6.9 (315)	28.1 ​± 6.5 (191)	.02
Medical history				
History of tobacco use	63.2 (449/710)	62.2 (191/307)	58.0 (109/188)	.42
Hypertension	90.5 (654/723)	93.3 (292/313)	94.8 (182/192)	.08
Dyslipidemia	78.2 (561/717)	80.9 (254/314)	84.8 (162/191)	.12
Diabetes	54.1 (391/723)	58.3 (183/314)	59.4 (114/192)	.27
Peripheral vascular disease	17.0 (122/717)	27.4 (85/310)	33.2 (63/190)	<.001
Chronic pulmonary disease	21.7 (155/715)	24.0 (75/313)	23.8 (45/189)	.66
Stroke/TIA	14.9 (107/719)	18.9 (59/312)	22.5 (43/191)	.03
Renal insufficiency	22.2 (160/721)	35.9 (112/312)	61.6 (117/190)	<.001
eGFR^a^, mL/min/1.73 m^2^	76.1 ​± 23.2 (566)	61.0 ​± 21.6 (257)	48.3 ± ​20.0 (137)	<.001
On dialysis	18.8 (30/160)	27.7 (31/112)	42.7 (50/117)	<.001
Prior myocardial infarction	36.7 (255/694)	42.9 (132/308)	50.3 (92/183)	.002
Prior coronary artery bypass grafting	8.9 (64/722)	18.5 (58/314)	30.2 (58/192)	<.001
Heart failure	55.4 (399/720)	63.9 (198/310)	73.3 (140/191)	<.001
Left ventricular ejection fraction, %	36.7 ​± 15.9 (503)	33.4 ​± 14.8 (260)	28.6 ± 15.3 (177)	<.001
Atrial fibrillation	33.9 (19/56)	38.1 (16/42)	43.8 (7/16)	.76
Indication for PCI				
Acute coronary syndrome	48.3 ​(305/631)	68.7 ​(193/281)	67.4 ​(116/172)	<.001
Urgent PCI	42.6 (310/728)	60.1 (190/316)	68.4 (132/193)	<.001
Number of diseased vessels				
1	12.5 ​(90/720)	10.2 ​(32/314)	8.3 ​(16/192)	.21
2	31.4 (226/720)	30.3 (95/314)	28.6 ​(55/192)	.75
3	55.4 ​(399/720)	55.4 ​(174/314)	62.0 ​(119/192)	.24
Left main disease	55.0 ​(398/723)	61.5 ​(193/314)	67.4 ​(128/190)	.004
Number of vessels treated				.92
1	29.2 (199/681)	27.8 (78/281)	29.1 (53/182)	.90
2	45.1 (307/681)	45.2 (127/281)	42.3 (77/182)	.78
3	25.7 (175/681)	27.0 (76/281)	28.6 (52/1825)	.72
Pre-PCI SYNTAX Score	27.2 ± ​12.2 (486)	27.8 ​± 12.3 (226)	31.4 ± ​13.2 (138)	.002
Post-PCI SYNTAX Score	6.5 ± 8.3 (482)	6.3 ± 8.2 (221)	7.7 ± 8.3 (137)	.27
Pre-PCI Ischemia Jeopardy Score	8.7 ​± 2.2 (566)	8.9 ± ​2.1 (258)	9.2 ​± 2.1 (151)	.04
Post-PCI Ischemia Jeopardy Score	1.9 ± 2.2 (562)	1.9 ± 2.2 (253)	2.1 ± 2.0 (150)	.49

Data are presented as mean ± SD (n) or % (n/N), where applicable.

eGFR, estimated glomerular filtration rate; PCI, percutaneous coronary intervention; STS, Society of Thoracic Surgeons; SYNTAX, Synergy Between Percutaneous Coronary Intervention with Taxus and Cardiac Surgery; TIA, transient ischemic attack.

a eGFR was calculated using 2021 Chronic Kidney Disease Epidemiology Collaboration (CKD-EPI) creatinine equation.

### Admission and procedural characteristics

Procedure duration and total length of hospitalization increased with higher STS category, whereas intraprocedural complication rates were comparable across groups. In contrast, adverse events—cardiac arrest, cardiogenic shock, and acute kidney injury—were more frequent in subjects with higher STS score. LM disease was more prevalent in the high-STS cohort than in the intermediate and low cohorts (67.4% vs 61.5% vs 55.0%; overall *P* = .004). Pre-PCI SYNTAX scores rose with increasing STS, while post-PCI SYNTAX scores were similar across strata (Tables 1 and 2).

**Table 2:** Immediate PCI-related complications and in-hospital adverse events according to STS risk score

	STS risk score			Overall *P* value
	Low (n = 728)	Intermediate (n = 316)	High (n = 193)	
Duration of index PCI procedure, h	1.9 ± 1.0 (n = 674)	2.1 ± 1.1 (n = 304)	2.3 ± 1.2 (n = 185)	.0008
Duration of hospitalization, d	4.0 ​​[1.0-9.0] (n = 722)	7.0 ​[4.0-12.0] (n = 315)	8.0 ​(4.0-14.0) (n = 187)	<.001
PCI-related complications^a^	4.6 (17/369)	4.7 (20/424)	2.2 (4/179)	.34
No reflow	6.3 (2/32)	0.0 (0/10)	5.8 (3/52)	.62
Abrupt closure	6.3 (2/32)	0.0 (0/10)	3.8 (2/52)	.52
Dissection	15.6 (5/32)	10.0 (1/10)	15.4 (8/52)	.82
Distal embolization	3.1 (1/32)	0.0 (0/10)	1.9 (1/52)	.73
Perforation	28.1 (9/32)	40.0 (4/10)	32.7 (17/52)	.67
Adverse events				
Pericardial effusion requiring pericardiocentesis	1.2 (9/727)	0.9 (3/316)	1.0 (2/193)	.91
Cardiac arrest	0.8 (6/727)	2.8 (9/316)	5.7 (11/193)	<.001
Cardiogenic shock	1.4 (10/727)	1.6 (5/316)	6.2 (12/193)	.0002
Ventricular arrhythmia	1.1 (8/727)	1.3 (4/316)	4.1 (8/193)	.010
AKI (stage 2 or 3)	2.9 (21/727)	4.7 (15/316)	8.8 (17/193)	.001
Life-threatening/disabling/major bleeding (BARC ≥3a)	2.1 (15/727)	3.2 (10/316)	3.1 (6/193)	.49
Life-threatening/disabling/major bleeding (BARC ≥3a) excluding dialysis patients^b^				
Hemolysis	0.4 (3/727)	1.9 (6/316)	2.6 (5/193)	.01
Thrombocytopenia	0.4 (3/727)	0.9 (3/316)	2.6 (5/193)	.02
Anemia requiring transfusion	5.2 (38/727)	7.9 (25/316)	17.1 (33/193)	<.001
Vascular/cardiac structural complication requiring surgery/re-intervention	0.4 (3/727)	0.3 (1/316)	1.0 (2/193)	.48
Access site-related hematoma	5.1 (37/727)	12.0 (38/316)	8.3 (16/193)	.004

Data are presented as mean ± SD, median [IQR], or % (n/N).

AKI, acute kidney injury; BARC, Bleeding Academic Research Consortium; PCI, percutaneous coronary intervention; STS, Society of Thoracic Surgeons.

a PCI-related complications are defined as the composite of no reflow, abrupt closure, dissection, distal embolus and perforation during PCI.

b Sensitivity analysis was conducted excluding dialysis patients and using the same statistical methods for the entire cohort, as explained in Methods.

### Major adverse cardiovascular and cerebrovascular events

**Figure 2:**
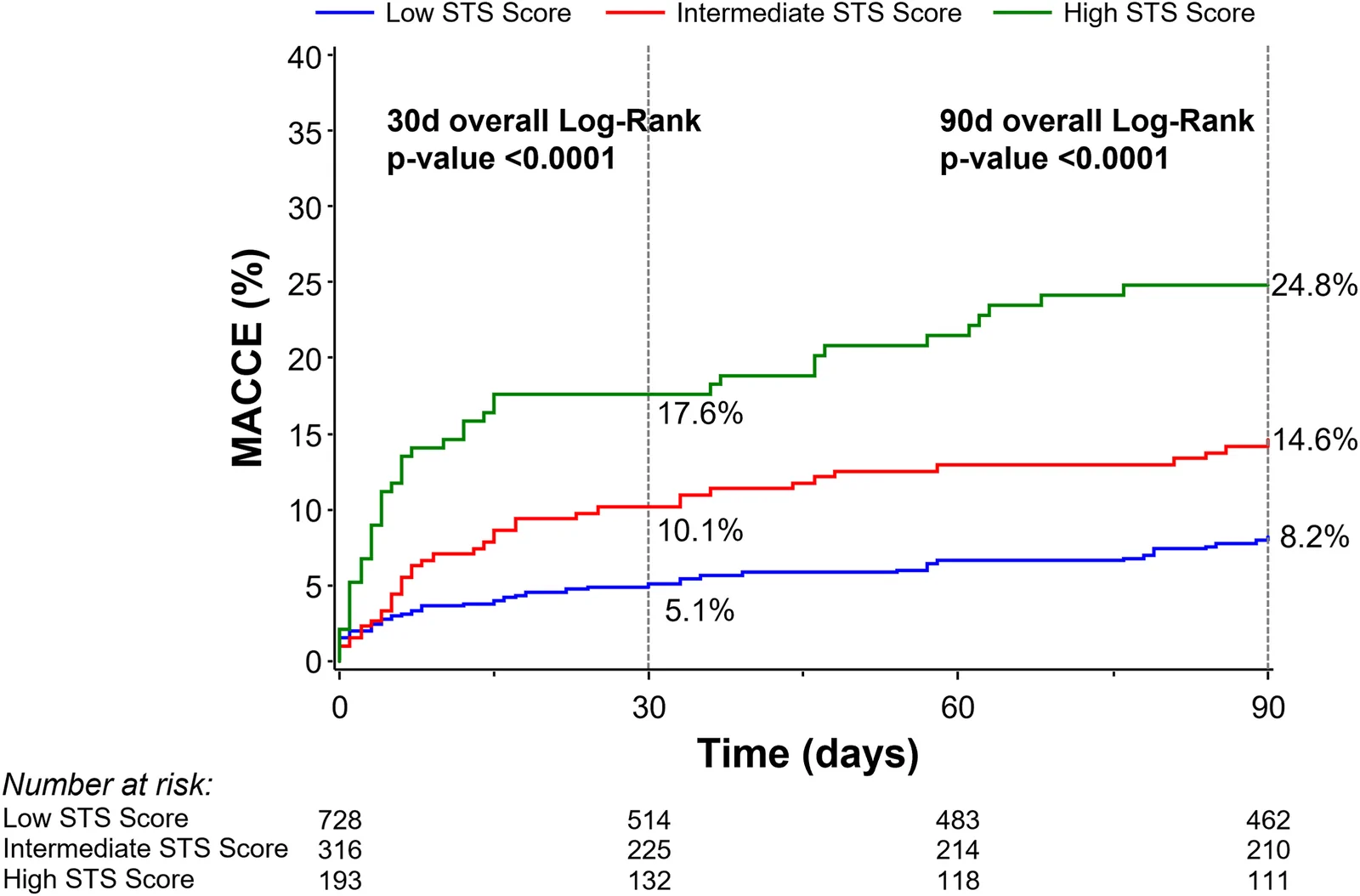
**Kaplan–Meier curves for 30 and 90 days MACCE stratified by STS Score.** MACCE, major adverse cardiovascular and cerebrovascular events; STS, Society of Thoracic Surgeons.

**Figure 3:**
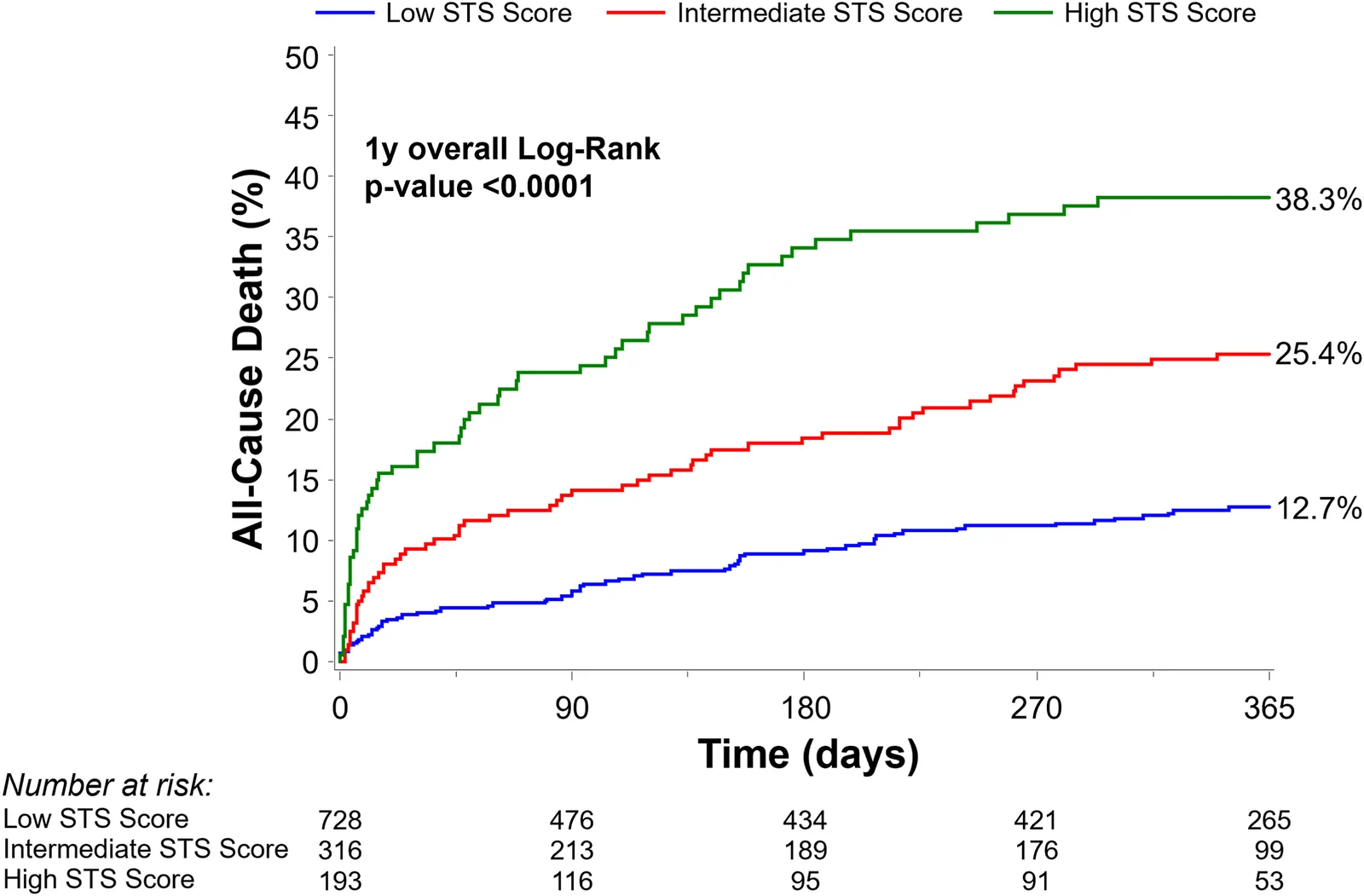
**Kaplan–Meier curves for all-cause mortality at 1 year stratified by STS Score.** STS, Society of Thoracic Surgeons.

Kaplan–Meier curves demonstrated an increase in MACCE across STS risk groups. At 30 days, MACCE were 5.1% in the low-STS group, 10.1% in the intermediate group, and 17.6% in the high group (overall *P* < .001). This was driven primarily by mortality (30-day death: 3.5% vs 8.6% vs 16.8%; overall *P* < .001), whereas 30-day myocardial infarction, stroke/transient ischemic attack, and repeat revascularization did not differ significantly between groups (Table 3). This separation in MACCE persisted through 90 days (8.2% vs 14.6% vs 24.8%; overall *P* < .001; Figure 2; Table 3). At 1 year, all-cause mortality increased progressively across STS categories, with Kaplan–Meier event rates of 12.7% in the low-risk group, 25.4% in the intermediate-risk group, and 38.3% in the high-risk group (overall log-rank *P* < .001). The curves separated early after the index procedure and continued to diverge throughout follow-up (Figure 3).

**Table 3:** Thirty-day and 90-day major adverse cardiac and cerebrovascular events and 1-year mortality according to STS Risk Score

	STS risk score			Overall *P* value
	Low	Intermediate	High	
30-Day MACCE^a^	5.1 (32)	10.1 (28)	17.6 (32)	<.001
Death	3.5 ​(21)	8.6 ​(23)	16.8 ​(30)	<.001
Noncardiovascular	0.2 (1)	0.8 ​(2)	2.5 ​(4)	.007
Cardiovascular	3.3 (20)	7.8 (30)	14.6 (26)	<.001
Myocardial infarction	1.0 ​(6)	2.7 (7)	5.0 (9)	.002
Stroke/TIA	1.2 ​(8)	2.3 ​(7)	1.6 ​(3)	.40
Repeat revascularization	0.4 ​(2)	1.2 (3)	1.3 ​(2)	.29
90-Day MACCE^a^	8.2 ​(48)	14.6 ​(39)	24.8 ​(43)	<.001
Death	5.4 ​(31)	13.4 ​(35)	22.0 ​(38)	<.001
Noncardiovascular	0.4 ​(2)	1.7 ​(4)	2.5 (4)	.03
Cardiovascular	5.1 ​(29)	11.9 ​(31)	20.0 ​(34)	<.001
Myocardial infarction	2.4 ​(13)	4.0 ​(10)	7.4 ​(12)	.004
Stroke/TIA	1.2 ​(8)	2.8 ​(8)	1.6 ​(3)	.24
Repeat revascularization	2.0 (3)	1.2 (10)	4.5 ​(6)	.11
1-Year mortality	12.7 (66)	25.4 (63)	38.3 (62)	<.001

Values are Kaplan–Meier event rates (n of events). Event rates are Kaplan-Meier event rates and compared by the log-rank test.

MACCE, major adverse cardiovascular and cerebrovascular events; STS, Society of Thoracic Surgeons; TIA, transient ischemic attack.

a MACCE is defined as the composite of all-cause death, myocardial infarction, stroke/TIA, and revascularization.

### Performance of the STS risk score

#### Discrimination (receiver operating characteristic analysis)

**Figure 4:**
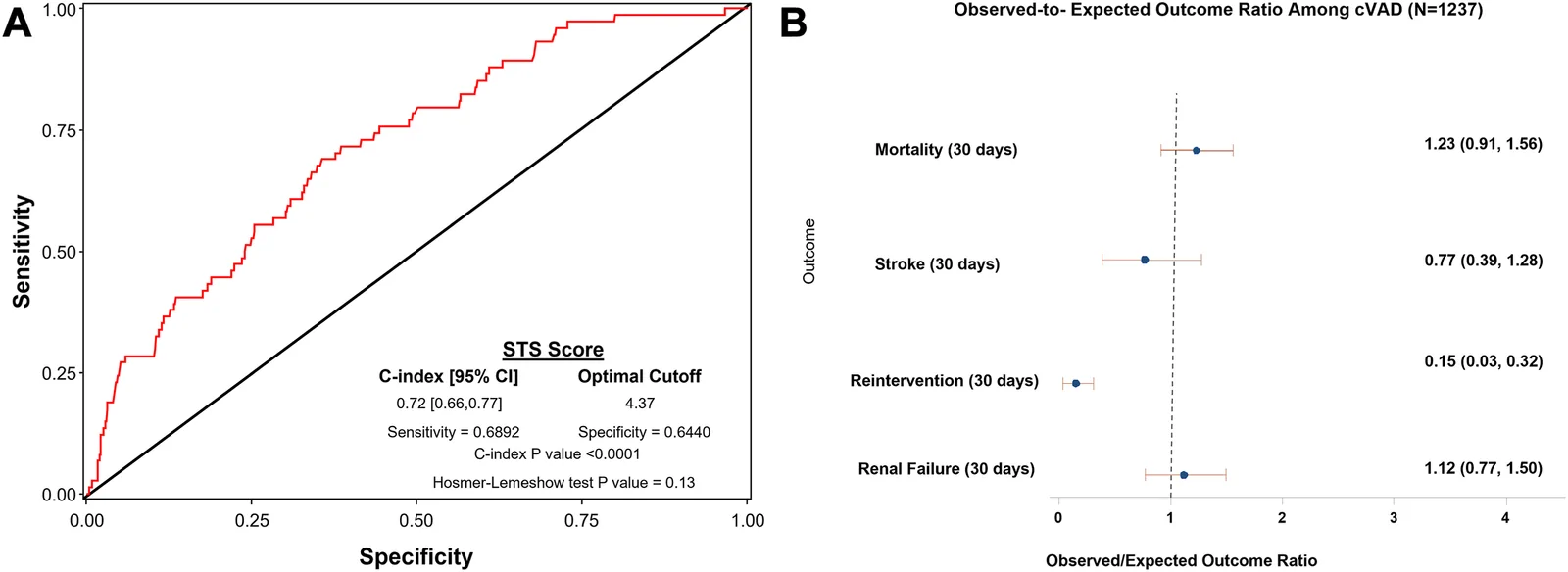
**STS performance.** (A) Receiver operating characteristic analysis of STS Score showing sensitivity and specificity at optimal cutoff. (B) Observed-to-expected ratio. STS, Society of Thoracic Surgeons.

The STS risk score demonstrated good discriminatory ability in this cohort, with a C-index of 0.72 (95% CI, 0.66-0.77; *P* < .001). This suggests that the model can fairly distinguish between patients who experienced adverse outcomes and those who did not. At the optimal cutoff of 4.37, the STS score achieved a sensitivity of 68.9% and specificity of 64.4%, indicating a reasonable balance between correctly identifying high-risk patients and minimizing false positives. Importantly, calibration was acceptable, as reflected by a nonsignificant Hosmer–Lemeshow test (*P* = .13), suggesting overall agreement between predicted and observed outcomes at the population level. Figure 4A illustrates the receiver operating characteristic curve for the STS score, demonstrating good discrimination for 30-day mortality across the range of predicted risk.

#### Calibration (O/E ratios)

When examining endpoint-specific calibration, the STS score showed variable performance across outcomes, as illustrated in Figure 4B. For 30-day mortality, the model slightly underestimated risk with an O/E ratio of 1.23 (95% CI, 0.91-1.56), indicating that more deaths occurred than predicted. Similarly, renal failure at 30 days was underestimated (O/E ratio, 1.12; 95% CI, 0.77-1.50). In contrast, the STS score overestimated stroke (O/E ratio, 0.77; 95% CI, 0.39-1.28), and revascularization was markedly overestimated (O/E ratio, 0.15; 95% CI, 0.03-0.32). These findings highlight that while the STS score maintains reasonable global discrimination and calibration, it does not uniformly predict all adverse outcomes, with a tendency to overestimate procedural complications and underestimate mortality risk in this high-risk PCI population.

## Discussion

Risk prediction in patients undergoing HRPCI remains an evolving, clinically important domain that underpins risk assessment and shared decision making. As the HRPCI population grows with older age, more complex anatomy, greater comorbidity burden, and delayed presentation, tailored tools are increasingly needed. A dedicated risk score for Impella-supported HRPCI was recently published from the cVAD registry and showed encouraging discrimination; however, it has not yet been externally validated.^14^ Importantly, patients selected for Impella-supported HRPCI often exhibit anatomical complexity highly similar to those treated with CABG—for example, LM or 3-vessel disease, diffuse calcification, and complex bifurcations—despite being poor surgical candidates due to high-risk morbidity, mortality, and poor functional recovery. This overlap in coronary disease complexity provides a strong rationale to examine whether models developed for surgical cohorts can inform risk in this setting. Accordingly, our study evaluates the performance of the STS risk score when applied to HRPCI supported with pLVADs, with the aim of informing preprocedural patient counseling and procedural planning and enabling more consistent benchmarking across centers. Our analysis confirmed the STS score effectively differentiated risk strata—patients with higher STS values experienced worse outcomes than those with lower scores. The model demonstrated good discrimination (C-index 0.72) and acceptable overall calibration (Hosmer–Lemeshow *P* = 0.13) but tended to underestimate mortality (O/E 1.23) and renal failure (1.12) while overestimating stroke (0.77) and revascularization (reoperation) (0.15) (Central Illustration; Figure 4).

**Central Illustration:**
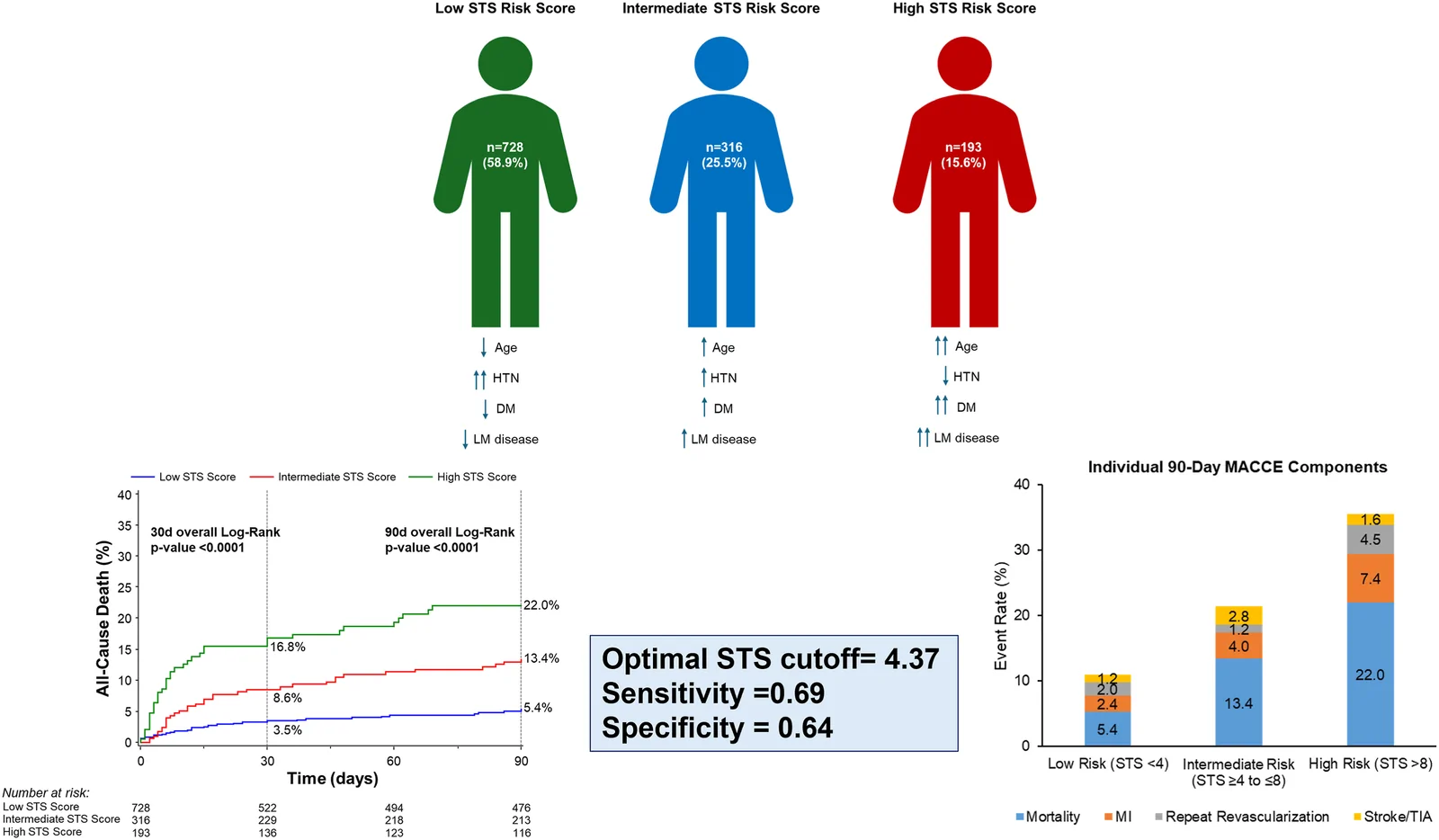
**The association between increasing STS risk score categories and worse 90-day outcomes in patients undergoing high-risk PCI.** DM, diabetes mellitus; HTN, hypertension; LM, left main; MACCE, major adverse cardiovascular and cerebrovascular event; MI, myocardial infarction; STS, Society of Thoracic Surgeons; TIA, transient ischemic attack.

The STS risk score was originally developed as a robust and widely adopted tool to estimate 30-day mortality and major complications after CABG. It was derived from the large, nationwide STS Adult Cardiac Surgery Database, which was established in 1989, integrating a broad range of clinical comorbidities, demographics, and operative variables to quantify perioperative risk in a reproducible and objective manner.^15^ Since its introduction, the STS model has undergone extensive external validation across diverse surgical populations, consistently demonstrating good calibration and discriminatory performance in real-world practice.^18^ Moreover, the model has been refined and updated to reflect advances in surgical techniques and evolving patient characteristics, with tailored risk models now available for specific procedures such as isolated CABG, valve surgery, and combined operations.^16^ However, by design, the STS model was not intended for patients undergoing PCI, and importantly, the derivation cohort did not include those deemed prohibitive risk patients for surgery—a group that now commonly undergoes HRPCI. This context is essential for interpreting the findings of our analysis.

In the PROTECT III cohort, which enrolled patients systematically deemed high-risk, and the majority of whom were evaluated and found unsuitable for surgery,^7^ the STS score demonstrated good discriminatory ability for short-term outcomes. Its performance was superior to that reported for other established models, such as the National Cardiovascular Data Registry and Complex High-Risk Indicated PCI risk scores, and was comparable to the recently published novel HRPCI risk score.^14^ Specifically, the recently developed HRPCI-specific model demonstrated an area under the curve of 0.75 for 30-day mortality, only modestly higher than the area under the curve of 0.72 observed for the STS score in the present analysis.^14^ This likely reflects the fact that the HRPCI model was derived specifically in patients undergoing pLVAD-supported HRPCI and incorporates variables particularly relevant to this setting, whereas the STS score was developed in surgical populations. Nevertheless, the relatively similar performance of the 2 models suggests that the STS score may still provide useful prognostic information in the absence of an externally validated HRPCI-specific tool. This may be explained by the fact that the STS model incorporates a broad range of clinical variables and comorbidities, thereby more fully capturing the baseline complexity of this patient population. However, since this risk score was not developed for this high-risk population, we observed bidirectional miscalibration: mortality and renal failure were underestimated, while neurological and surgical complications, such as stroke and reoperation, were overestimated. The finding that observed mortality exceeded predicted values highlights the particularly high-risk nature of HRPCI patients, whose baseline frailty, complex coronary anatomy, and hemodynamic vulnerability exceed the parameters captured by surgical cohorts.

Notably, a recent analysis of patients within this cohort who were formally evaluated and declined for surgery demonstrated even poorer outcomes than the overall population,^7^ further emphasizing the critical role of clinical judgment and multidisciplinary evaluation—factors not reflected in existing risk models. These qualitative assessments, although not easily incorporated into quantitative models, may capture aspects of risk particularly relevant to HRPCI.

Our findings are consistent with prior analyses from the PROTECT II trial, in which 6 established surgical and PCI-based risk models, including the STS mortality and morbidity/mortality models, EuroSCORE, and regional PCI scores, were evaluated in mechanically supported HRPCI. In that study, surgical models such as STS mortality, STS morbidity/mortality, and EuroSCORE consistently underpredicted mortality, whereas PCI-based models like the Mayo PCI and New York State PCI scores tended to overpredict risk. Despite these opposing directions of miscalibration, all models demonstrated only modest discrimination (C-statistic, 0.54-0.62) and poor calibration.^19^ These results parallel our observations in PROTECT III and reinforce the concept that conventional surgical and PCI scores fail to capture the unique risk profile of HRPCI patients, many of whom are surgically ineligible, frail, and hemodynamically unstable. Both the PROTECT II and PROTECT III experiences therefore highlight the urgent need for recalibrated or dedicated HRPCI-specific risk models that more accurately reflect this contemporary patient population.

Improving calibration of existing models and incorporating clinical assessment may represent important next steps in refining risk prediction for HRPCI. Adding these dimensions to an already familiar tool could create a more accurate and clinically meaningful score. By enhancing calibration and integrating physician’s clinical judgment, the STS framework could evolve into a standardized platform applicable to both surgeons and interventional cardiologists, facilitating more consistent decision making and aligning perspectives across disciplines.

At the same time, we should also strive to validate the recently developed HRPCI-specific risk score in external populations of pLVAD-supported patients beyond the cVAD registry. Such efforts are essential to confirm generalizability and to advance the development of truly dedicated risk assessment models in this high-risk cohort. Beyond individual patient care, a standardized and validated model has the potential to unify reporting and benchmarking across institutions. The STS score is already widely recognized and embedded in surgical practice; adapting it for HRPCI could establish a common language for evaluating outcomes, guiding procedural planning, and assessing new devices or strategies. Its familiarity would also support patient counseling, helping clinicians contextualize HRPCI risks relative to surgical or medical therapy. Until HRPCI-specific tools undergo broad validation, the STS model—augmented by recalibration, clinical input, and parallel efforts to externally validate novel scores—offers a pragmatic bridge toward more robust and universally accepted risk assessment.

In conclusion, the STS risk score provides reasonable discrimination in Impella-supported HRPCI but does not accurately calibrate outcomes, particularly underestimating mortality. This highlights the uniquely high-risk nature of the PROTECT III cVAD population and underscores the need for recalibration, incorporation of clinical judgment, and need for external validation of HRPCI-specific tools to create standardized frameworks for decision making and benchmarking across institutions.

## CRediT authorship contribution statement

**Batla Falah:** Conceptualization, Writing – original draft, Writing – review & editing. **Julia Thompson:** Formal analysis. **Yousuf Shah:** Writing – original draft. **Tayyab Shah:** Writing – original draft. **David J. Cohen:** Writing – original draft. **Guido Ascione:** Writing – original draft. **Pedro Melo:** Writing – original draft. **Björn Redfors:** Writing – original draft. **Mir Babar Basir:** Writing – original draft. **Wayne B. Batchelor:** Writing – original draft. **Alejandro Lemor:** Writing – original draft. **Alexander G. Truesdell:** Writing – original draft. **Cindy L. Grines:** Writing – original draft. **William W. O’Neill:** Supervision, Writing – original draft.

## Peer review statement

Associate Editor Cindy L. Grines had no involvement in the peer review of this article and has no access to information regarding its peer review. Full responsibility for the editorial process for this article was delegated to Associate Editor Sandeep Nathan.

## Declaration of competing interest

Yousuf Shah is the founder of Meducator LLC, which facilitated some programming analyses. Tayyab Shah has received speaking honorariums from Abiomed. David J. Cohen reports institutional research support from Abbott, Edwards Lifesciences, Boston Scientific, Philips, Corvia Medical, Zoll Medical, CathWorks, and ANCORA and consulting income from Abbott, Edwards Lifesciences, Boston Scientific, Medtronic, and Elixir Medical. Björn Redfors reports consultant fees from Pfizer and Boehringer Ingelheim. Mir Babar Basir reports consultant fees from Abiomed, Boston Scientific, Chiesi, and Zoll. Wayne B. Batchelor has received consulting fees from Boston Scientific, Edwards Lifesciences, Chiesi, and Medtronic. Alejandro Lemor reports speaker fees from Abiomed. Alexander G. Truesdell reports speaking/consultant fees from Abiomed, Getinge, Shockwave Medical, and Zoll. Cindy L. Grines reports participation on the advisory boards for Philips and Abiomed. William W. O’Neill reports grant/research support from St. Jude Medical, Edwards Lifesciences, and Abiomed; consulting fees/honoraria from Medtronic and Abiomed; and major stock shareholder/equity in Synecor, Accumed, Neovasc, Tendyne, and Mitralign. All other reported no financial interests.
